# Sulfur as a central integrator of plant–microbe interactions: from nutrient cycling to immune signalling and microbiome assembly

**DOI:** 10.1093/jxb/erag186

**Published:** 2026-04-17

**Authors:** Büsra Elkatmis, Gözde Merve Türksoy, Enrique Rodríguez, Bilal Rahmoune, Anna Koprivova, Stanislav Kopriva

**Affiliations:** Institute for Plant Sciences, Centre of Excellence on Plant Sciences, University of Cologne, D-50674 Cologne, Germany; Institute for Plant Sciences, Centre of Excellence on Plant Sciences, University of Cologne, D-50674 Cologne, Germany; Max Planck Institute for Plant Breeding Research, Carl-von-Linne-Weg 10, D-50829 Cologne, Germany; Institute for Plant Sciences, Centre of Excellence on Plant Sciences, University of Cologne, D-50674 Cologne, Germany; Max Planck Institute for Plant Breeding Research, Carl-von-Linne-Weg 10, D-50829 Cologne, Germany; Institute for Plant Sciences, Centre of Excellence on Plant Sciences, University of Cologne, D-50674 Cologne, Germany; Institute for Plant Sciences, Centre of Excellence on Plant Sciences, University of Cologne, D-50674 Cologne, Germany; Institute for Plant Sciences, Centre of Excellence on Plant Sciences, University of Cologne, D-50674 Cologne, Germany; Bielefeld University, Germany

**Keywords:** Camalexin, glucosinolates, microbiome, plant defense, plant growth-promoting bacteria, S-containing metabolites, sulfur, volatiles

## Abstract

Sulfur is an essential macronutrient that underpins plant growth, stress resilience, and immunity. Beyond its role in primary metabolism, sulfur is incorporated into a diverse array of secondary metabolites that mediate plant–microbe interactions. In this review, we summarize current knowledge on how microbial sulfur metabolism contributes to plant sulfur nutrition and how plant-derived sulfur-containing compounds shape microbial community assembly and disease outcomes. Microorganisms mobilize organic sulfur in soils through sulfatase activity, volatile sulfur production, and sulfoquinovose degradation, thereby enhancing plant sulfur availability, particularly under limiting conditions. Conversely, plants deploy sulfur-rich metabolites, including volatile organic compounds, glucosinolates, and the phytoalexin camalexin, to restrict pathogens, modulate beneficial associations, and structure rhizosphere communities. These compounds act not only as antimicrobial agents but also as ecological filters that balance defense with microbiome homeostasis. Emerging evidence indicates that sulfur availability and metabolic flux influence the composition and function of plant-associated microbiota, linking primary nutrient assimilation to immune regulation. By integrating insights from sulfur biochemistry, microbial ecology, and plant immunity, we highlight sulfur metabolism as a central node in plant–microbe interactions. Understanding the dynamic exchange of sulfur between plants and their microbiota will be essential for improving crop resilience and sustainable nutrient management in sulfur-limited agricultural systems.

## Teaser

This review summarizes current knowledge on the role of sulfur compounds in plant interactions with pathogens and plant growth-promoting bacteria.

## Introduction

Plants inhabit complex microbial ecosystems in which beneficial, commensal, and pathogenic microorganisms co-exist (Bulgarelli *et al*., 2013). Beneficial microorganisms contribute to plant performance by improving nutrient bioavailability (Hacquard *et al*., 2015), protecting plants against adverse environmental conditions (de Zelicourt *et al*., 2018) and suppressing pathogens (Duran *et al*., 2018; Carrion *et al*., 2019). In contrast, harmful microbes cause a large variety of plant diseases, with significant impacts on crop production (Strange and Scott, 2005). Plants evolved diverse mechanisms to communicate with the microbiota, selectively attracting beneficial organisms while defending against harmful ones. Many of these mechanisms rely on plant metabolites, several of which can fulfill both of these functions (reviewed in Sasse *et al*., 2018; Jacoby *et al*., 2020; Somssich *et al*., 2025). Among mineral nutrients, sulfur (S) occupies a uniquely versatile and often underappreciated position in these regulatory networks (Takahashi *et al*., 2011; Mukherjee *et al*., 2025a).

S is indispensable for primary metabolism as a constituent of cysteine, methionine, coenzymes, prosthetic groups, and the central redox buffer glutathione. The chemical flexibility and redox versatility of S, namely its ability to cycle between multiple oxidation states, underlies its central role in redox regulation and stress adaptation (Jacob and Anwar, 2008). Plants take up S as inorganic sulfate, reduce it, and incorporate it as sulfide into *O*-acetylserine (OAS), to form Cys, the first product of sulfate assimilation (Takahashi *et al*., 2011). Cys is incorporated into proteins, converted into Met and glutathione (GSH), or serves as an S donor for the synthesis of numerous S-containing metabolites, that contribute to innate immunity (Rausch and Wachter, 2005).

Bacterial S metabolism is likewise critical for plant interactions. For example, sulfation is required for the function of many Nod factors in *Rhizobium* symbiosis (Schwedock and Long, 1990), and sulfate assimilation genes *CysH* and *CysM* are necessary for the plant growth-promoting (PGP) effect of *Pseudomonas fluorescens* strain SS101 (Cheng *et al*., 2017). Similarly, disruption of S assimilation pathway reduces virulence in multiple fungal and bacterial pathogens (Shen *et al*., 2002; Zhao *et al*., 2022; Lee *et al*., 2023).

Recent advances in synthetic community approaches, isotope tracing, and multi-omics analyses have begun to reveal how tightly coordinated plant and microbial S metabolism governs both mutualistic and antagonistic interactions. Yet, critical mechanistic questions remain unresolved, particularly regarding S fluxes between partners, perception of S-containing volatiles, and ecological relevance under field conditions. Here, we summarize current knowledge on (i) microbial contributions to plant S nutrition, (ii) S-containing volatile organic compounds (VOCs) as mediators of long-distance plant–microbe communication, (iii) S-dependent plant defense metabolites in pathogen resistance and microbiome assembly, and (iv) the role of S metabolism in beneficial interactions and abiotic stress tolerance. We propose that S metabolism acts as a central integrator linking nutrient cycling, immune signaling, redox regulation, and microbial community dynamics. Since the most intensively studied S-containing specialized metabolites, glucosinolates and camalexin, are restricted to the Brassicales, this family, including the model plant *Arabidopsis thaliana*, is over-represented in the examples discussed. Nevertheless, many underlying mechanisms are conserved across plant species.

## Beneficial impact of microbial sulfur metabolism on plants

### Microbes in plant sulfur nutrition

In nature, S can be transformed between organic and inorganic forms and multiple oxidation states through both natural and anthropogenic processes (Z. Zhou *et al*., 2025). Biogenic transformations are major drivers of the global S cycle and have played essential roles in the evolution of life on Earth (Mateos *et al*., 2023). S is ubiquitous in terrestrial and marine environments, as well as in the atmosphere, thereby influencing climate dynamics (Z. Zhou *et al*., 2025). The S cycle has changed substantially due to anthropogenic activities. Since the industrial revolution, increasing S emissions led to acid rain and severe health and environmental problems. Following implementation of the Clean Air Act in 1970, S depositions decreased significantly and S has again become limiting in many agricultural systems (Hinckley *et al*., 2020). Because microorganisms drive key S transformations, biological S metabolism strongly impacts global S cycling, plant productivity, and ultimately human health (Mukherjee *et al*., 2025a).

Although plants take up S primarily as inorganic sulfate, most soil S occurs in organic forms that are not directly accessible to plants. Microorganisms can utilize these organic S sources and mineralize them, thereby providing S to plants (Kertesz and Mirleau, 2004). Three major pathways for mineralization of organic S are well established: (i) hydrolysis of sulfates and sulfonates by sulfatases (Kertesz *et al*., 2007); (ii) synthesis of the volatile compound dimethyl disulfide (DMDS) (Meldau *et al*., 2013); and (iii) metabolism of sulfoquinovose, the headgroup of plant sulfolipids (Snow *et al*., 2021).

Sulfatases are hydrolytic enzymes that operate via a unique catalytic mechanism involving formylglycine, generated post-translationally from Cys or Ser residues (Dierks *et al*., 1999). These enzymes enable bacteria to desulfurize diverse organic sulfates and sulfonates, acquiring both carbon and S (Kertesz, 2000; Barbeyron *et al*., 2016). Sulfatase-producing bacteria are common in the rhizosphere, and their activity often contributes directly to the PGP effects (Kertesz and Mirleau, 2004). Indeed, loss of the ability to utilize organic sulfates in the PGP bacterium *Pseudomonas putida* S-313R abolished its PGP effect, highlighting sulfatase activity as a key determinant of sulfate availability to plants.

A well-characterized S-dependent mechanism of bacterial PGP activity, particularly under low S supply, is the production of DMDS (Fig. 1) by *Bacillus* sp. B55 (Meldau *et al*., 2013). This strain colonizes wild tobacco *Nicotiana attenuata* and enhances plant growth via volatile emissions. S derived from DMDS can be incorporated into the plant organic matter, as demonstrated by tracer experiments (Meldau *et al*., 2013; and see below). In contrast to sulfate mineralization and DMDS production from various S-containing substrates, bacterial sulfoquinovose degradation (sulfoglycolysis) begins with a defined substrate, sulfoquinovose (Fig. 1), yet the specific form of S made available to plants remain unclear (Snow *et al*., 2021). Sulfoquinovose is among the most abundant organic S compounds in soil, with an estimated annual production of 10 billion tons (Sharma *et al*., 2022). Bacteria metabolize sulfoquinovose as a carbon source via distinct pathways, releasing S in forms such as sulfate, sulfite, sulfolactate, or dihydroxypropane-sulfonate (Snow *et al*., 2021). Although the biochemical details of these pathways are increasingly understood (Burchill *et al*., 2025), their quantitative contribution to plant S nutrition remains to be determined.

**Fig. 1.**
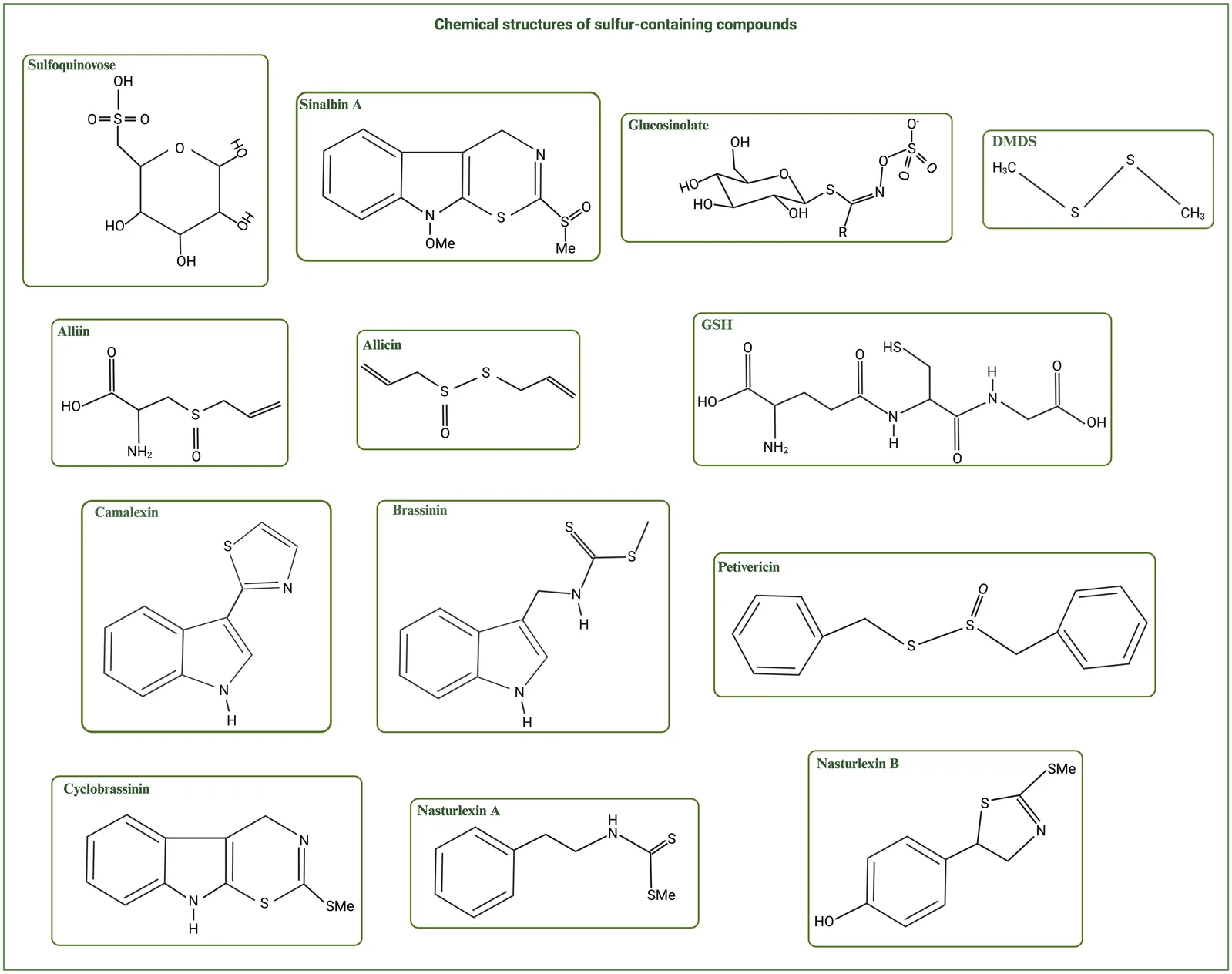
Representative chemical structures of key sulfur-containing compounds. The structures of sulfoquinovose, camalexin, brassinin, glucosinolates, dimethyl disulfide (DMDS), nasturlexin A, nasturlexin B, sinalbin A, alliin, allicin, cyclobrassinin, petivericin, and glutathione (GSH). Created in BioRender. Elkatmis, B. (2026) https://BioRender.com/yc1v6pp.

Beyond these defined pathways, numerous enzymatic reactions interconvert S compounds in the biosphere, including processes generating sulfide, oxidizing thiosulfate to sulfate, or producing dimethyl sulfide (Mukherjee *et al*., 2025a). Genes encoding these reactions are enriched in rhizosphere microorganisms compared with bulk soil, suggesting coordinated S metabolism between plants and microbes in the rhizosphere (Mukherjee *et al*., 2025a). Supporting this view, inoculation with mixed S-oxidizing and S-reducing bacteria increased nitrogen, phosphorus, and S accumulation in canola when combined with inorganic fertilizer (Aslam *et al*., 2025). Likewise, an 18-member synthetic rhizosphere bacterial community (SynCom) enhanced biomass of Arabidopsis and *Brassica rapa* var. *parachinensis* under S deficiency (Mukherjee *et al*., 2025b). This effect was attributed to extracellular GSH secreted by the microbiome, which alleviated S deficiency-induced oxidative stress and was confirmed by revealing that deletion of the GSH biosynthetic gene *gshA* abolished the PGP effect (Mukherjee *et al*., 2025b). However, for this and other microbial S transformations, ecological significance in natural ecosystems and potential for improving S use efficiency in the agricultural context remain to be elucidated.

### Sulfur-containing bacterial volatile organic compounds and their effects on plants

Among the diverse microbial S transformations in the rhizosphere, the emission of S-containing VOCs represents a particularly dynamic interface between microbial metabolism and plant physiology. VOCs play important roles in plant communication with both beneficial and pathogenic microorganisms (Weisskopf *et al*., 2021). Bacterial VOCs belong to several classes of chemical compounds, including ketones, terpenes, nitrogen-containing volatiles, aromatic compounds, pyrazines, and many S-containing compounds (Weisskopf *et al*., 2021). Among these, S-containing VOCs (S-VOCs) are particularly reactive due to the redox properties of S and can act over short and long distances. They are by-products of bacterial primary and secondary metabolism and are derived mainly from H_2_S and methanethiol (Schulz and Dickschat, 2007). Many volatiles and some of their functions have been reported, but a comprehensive inventory of VOCs and an understanding of their functions are still far from being complete (Kemmler *et al*., 2025). It is particularly crucial to characterize the functions and biosynthesis of additional VOCs, as they represent important alternatives to the use of fertilizers and pesticides (Thomas *et al*., 2020).

Numerous studies have identified bacterial S-VOCs with beneficial properties. One of the most well-known S-VOC is DMDS, which has been found as a dominant compound in the VOC bouquets of various bacterial species, including *Burkholderia ambifaria*, *Bacillus* sp. B55, and *Pseudomonas tolaasii* (Groenhagen *et al*., 2013; Meldau *et al*., 2013; Lo Cantore *et al*., 2015). Treatment with bacterial VOCs, as well as DMDS as a pure compound in low concentrations, can promote the growth of Arabidopsis and other plant species. Similarly, a study on 40 bacterial strains isolated from desert plants such as agaves and cacti, revealed that S-VOCs, such as DMDS, dimethyl trisulfide (DMTS), and 3-(methylthio)-1-propanol, were dominant compounds in their VOCs and promoted the growth of Arabidopsis and *Nicotiana benthamiana* (Fig. 2) (Camarena-Pozos *et al*., 2019). Furthermore, S-VOCs were also prevalent in VOC bouquets of a 16-member SynCom derived from Arabidopsis roots, with compounds such as DMDS, 2-methyl-5-(methylthio)furan, and 2,4-dithiapentane showing a PGP activity in a pure form (Türksoy *et al*., 2025). Additionally, DMDS and benzothiazole were among the VOCs responsible for PGP activities of several *Burkholderia* strains on *Malus domestica* (Zeng *et al*., 2025). However, while these pure compounds show promise as biofertilizers in laboratory settings, the application in real field conditions as growth stimulants poses challenges. Concentrations that promote growth or protect against pathogens in the laboratory may not have the same effects in the field, and could even be toxic at higher doses. For instance, DMDS was found to be toxic at higher concentrations in some studies (Lo Cantore *et al*., 2015), and the field application of methanesulfoxide as a fungicide was toxic to other organisms (Joller *et al*., 2020). Therefore, it is crucial to establish systems that closely mimic natural conditions when using these VOCs as growth stimulants.

**Fig. 2.**
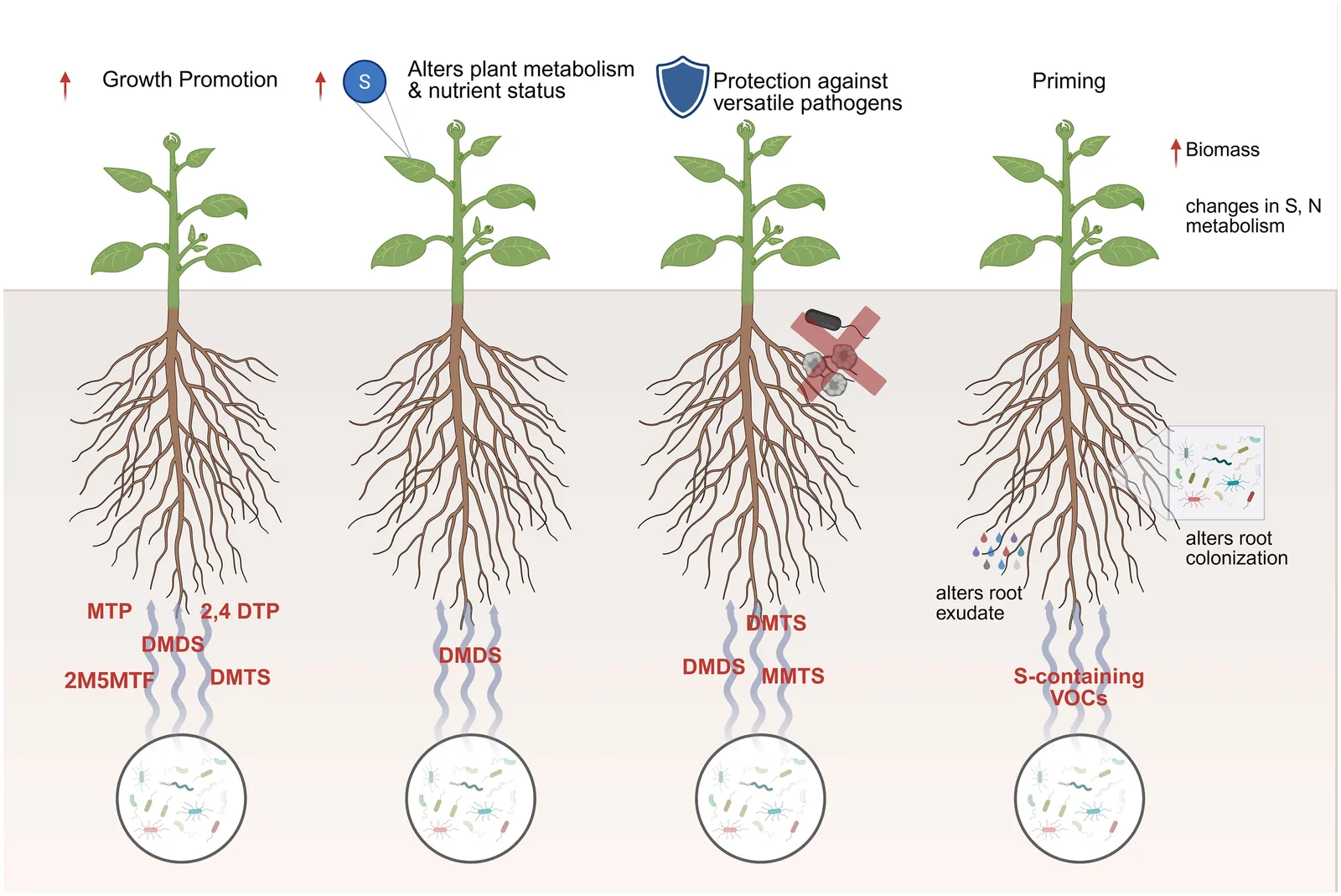
The effects of S-containing bacterial volatiles on plant growth, nutrition, defense, and physiological processes. From left to right: the first panel illustrates how S-containing bacterial volatiles—including dimethyl disulfide (DMDS), 2,4-dithiapentane (2,4-DTP), dimethyl trisulfide (DMTS), 3-(methylthio)-1-propanol (MTP), and 2-methyl-5-(methylthio)furan (2M5MTF)—promote growth in several plant species. The second panel shows that S-containing volatiles, particularly DMDS, can be directly utilized by plant metabolism as an environmental S source, leading to increased plant S nutrition. The third panel depicts the protective effects of S-containing volatiles—DMDS, DMTS, and *S*-methyl methanethiosulfonate (MMTS)—against various plant pathogens. The fourth panel highlights the priming functions of S-containing volatiles, which modulate multiple plant physiological processes, including increased plant biomass, alterations in S and nitrogen metabolism, changes in the composition of root-associated bacterial communities, and modifications of root exudate metabolite profiles. Created in BioRender. Türksoy, G.M. (2026) https://BioRender.com/u3v90b9.

To overcome such obstacles, researchers have explored using communities instead of single strains, or establishing more natural systems with soil. Türksoy *et al*. (2025) demonstrated that a SynCom produced growth-promoting VOCs dominated by S-containing metabolites. Removing high DMDS-producing strains or even all strains with PGP VOC cocktails did not diminish the overall PGP property of the SynCom (Türksoy *et al*., 2025). Soil-based systems, where bacteria and soil/pot-grown plants are separated by filters have also been employed; however, they may lead to side effects such as CO_2_ overaccumulation (Park *et al*., 2015; Tahir *et al*., 2017). Even though such systems are difficult to implement in field conditions, the priming effects of VOCs offer an encouraging and simple approach for applying either pure volatile compounds or whole volatile blends from bacteria and bacterial communities.

Despite the identification of volatiles that promote growth and stress tolerance, there is still a lack of understanding regarding how plants perceive them as signals, or whether they might be assimilated in plant metabolism. So far, no receptor has been identified for VOCs, except for ethylene (Bleecker *et al*., 1988). Among the S-VOCs, only the DMDS mode of action has been studied in detail. DMDS emitted by *Bacillus* sp. B55 can serve as an S nutrient source in *N. attenuata* (Meldau *et al*., 2013). ^35^S isotope labeling revealed the uptake and incorporation of S from VOCs into plant proteins. The PGP effect was clearly evident in sulfate-deficient conditions, so the ecological relevance of this process might be limited to S-deficient soils (Meldau *et al*., 2013). The growth-promoting effect of DMDS is dependent on OAS (thiol) lyase (OAS-TL), since loss of cytosolic or both plastidic and mitochondrial OAS-TL isoforms abolished the growth-promoting effect (Türksoy *et al*., 2025). This indicates that DMDS is reduced to methanethiol and in a reaction with OAS it is incorporated into *S*-methylcysteine, in analogy with the methionine degradation pathway. Indeed, metabolite analysis of shoots of Arabidopsis exposed to the community VOCs revealed an increase in *S*-methylcysteine concentration (Türksoy *et al*., 2025).

S-VOCs not only promote growth and S metabolism, but also influence nutrient homeostasis in different plant species (Fig. 2). For instance, volatiles emitted by *Serratia plymuthica* and *Fusarium culmorum* affected nutrient levels in *Zea mays* in a glass jar system (Martín-Sánchez *et al*., 2020). Analogously, bacterial VOCs enhanced iron acquisition, biomass, rhizosphere acidification, and chlorophyll content of *Medicago truncatula*, under both iron-limiting and non-limiting conditions. DMDS was detected in their volatile blend, along with another S-containing compound, DMTS (Orozco-Mosqueda Mdel *et al*., 2013).

Additionally, S-VOCs exhibit antimicrobial properties. For instance, S-VOCs produced by *Paraburkholderia graminis* contributed to the suppressiveness of soils against fungal pathogens (Carrion *et al*., 2018). DMDS protected tobacco and corn plants against *Botrytis cinerea* and *Cochliobolus heterostrophus* (Huang *et al*., 2012), and exhibited fungicidal activity against *Sclerotinia minor* (Tyagi *et al*., 2020). In addition, DMDS application on fungal-infected tomato plants induced overexpression of defense-related genes *PR1* and *PR5*, suggesting activation of the salicylic acid pathway (Tyagi *et al*., 2020). DMDS action is not restricted to fungal pathogens; it also inhibits growth of the oomycete *Phytophthora infestans* (De Vrieze *et al*., 2015). Among other S-VOCs, DMTS and *S*-methyl methanethiosulfonate (MMTS) were able to control potato infection by *P. infestans* (Chinchilla *et al*., 2019). Importantly, MMTS did not activate plant defenses prior to infection and directly inhibited the pathogen without being phytotoxic. However, its potential field application is limited, as it can be toxic to plants, bacteria, and nematodes, though it may still be used for direct oomycete control (Joller *et al*., 2020). DMTS has also been reported to reduce the incidence of *Botryosphaeria dothidea*, the causal agent of ring rot on apple fruit, and was effective in post-harvest protection. In addition to disease suppression, DMTS increased sugar content and vitamin C levels in apple fruit (Sun *et al*., 2022).

The protective action of VOCs can be attributed to their direct effects on pathogens and their ability to enhance plant innate immunity (Ryu *et al*., 2004; Erb *et al*., 2015). There is growing evidence that VOCs can prime plants to better withstand pathogen or herbivore attacks as well as to promote plant growth and yield. VOC blends containing S-VOCs have been particularly effective in priming plants for growth enhancement. For instance, Arabidopsis and *Lactuca sativa* treated with VOCs from a root-associated *Microbacterium* strain showed increased shoot biomass, flower number, and stem length upon transfer to soil. Transcriptomic analyses revealed that VOC treatment influenced plant S and nitrogen metabolism (Cordovez *et al*., 2018). Similarly, priming Arabidopsis with VOCs from a 16-member SynCom, which includes DMDS and many other S-VOCs, resulted in growth promotion and altered root colonization patterns (Türksoy *et al*., 2025). This priming affected root exudate composition, with an initial increase in exudation followed by a decrease. Furthermore, the concentration of S-containing defense compounds, indolic glucosinolates and camalexin, decreased, potentially to facilitate colonization (Türksoy *et al*., 2025). Interestingly, the plant-derived S-VOC, dipropyl disulfide, was also found to prime neighboring plants and influence root exudate composition, resulting in altered microbiome composition (Zhou *et al*., 2024).

These findings collectively suggest that bacterial S-VOCs, especially DMDS, play critical roles in plant–microbe interactions (Fig. 2). S-VOCs are emitted by various bacterial species and impact a wide range of plant species. While the biosynthesis of the VOCs is relatively well understood, the receptors in plants, signaling pathways, and detailed mechanisms of action remain largely unexplored. Discovering new bacterial VOCs with plant bioactivity is challenging due to issues with identification and availability of the compounds. Although DMDS may provide S for plant metabolism, this is not confirmed for other S-VOCs. It is also unclear if the positive effects observed in laboratory settings translate to natural ecosystems. Further research is needed to understand the mechanisms and potential agricultural uses of these S-VOCs.

## Plant sulfur-containing metabolites interacting with microbiota

### Role of plant sulfur-containing metabolites in pathogen defense

S has a long history of use as a phytosanitary agent dating back to ancient civilizations, such as the Sumerians and the Greeks. It is still used in modern pesticide formulations, such as fungicides, insecticides, acaricides, and nematicides (Umetsu and Shirai, 2020). The use of S in agriculture reflects the natural defense mechanisms of plants. Plants rely on S-containing compounds for their defense systems, reflected in the concept of S-induced resistance and S-enhanced defense (Rausch and Wachter, 2005; Bloem *et al*., 2015). These compounds, such as glucosinolates, S-containing phytoalexins, and S-VOCs, play a crucial role in plant immunity against a wide range of pathogens, parasites, and herbivores (Halkier and Gershenzon, 2006; Burow *et al*., 2008; Kunstler *et al*., 2020; Somssich *et al*., 2025). Their diverse structures and biosynthetic pathways make them powerful defense systems in a wide range of plant species.

Glucosinolates (Fig. 1), present in the Brassicales, are phytoanticipins, that are stored within plant tissue and act as pre-formed precursors of antimicrobial compounds. Tissue disruption or internal signaling pathways activate myrosinase-mediated hydrolysis of glucosinolates, generating bioactive breakdown products such as isothiocyanates, nitriles, and thiocyanates. These compounds provide defense against pathogens and herbivores (Halkier and Gershenzon, 2006; for details see below), and also play a crucial role in plant–insect chemical signaling and multitrophic interactions (Fig. 3) (Hopkins *et al*., 2009). Over 130 glucosinolate variants have been identified in plants (Blazevic *et al*., 2020), featuring a sulfated thioglucoside core linked to variable amino acid-derived side chains (Fig. 1). The chemical diversity of glucosinolates arises from side chain modifications that determine their biochemical properties and bioactivities of the degradation products (Halkier and Gershenzon, 2006). Glucosinolates are categorized into three groups based on the amino acids from which they are synthesized: aliphatic glucosinolates from Met, Val, Leu, Ile, and Ala, indolic glucosinolates from Trp, and benzenic glucosinolates from Phe or Tyr (Blazevic *et al*., 2020). The synthesis of glucosinolates involves three major stages—chain elongation of some precursor amino acids, core structure synthesis, and secondary modifications. Biosynthesis is tightly linked to S metabolism, providing S for the core glucosinolate structures (Mugford *et al*., 2009; Sonderby *et al*., 2010).

**Fig. 3.**
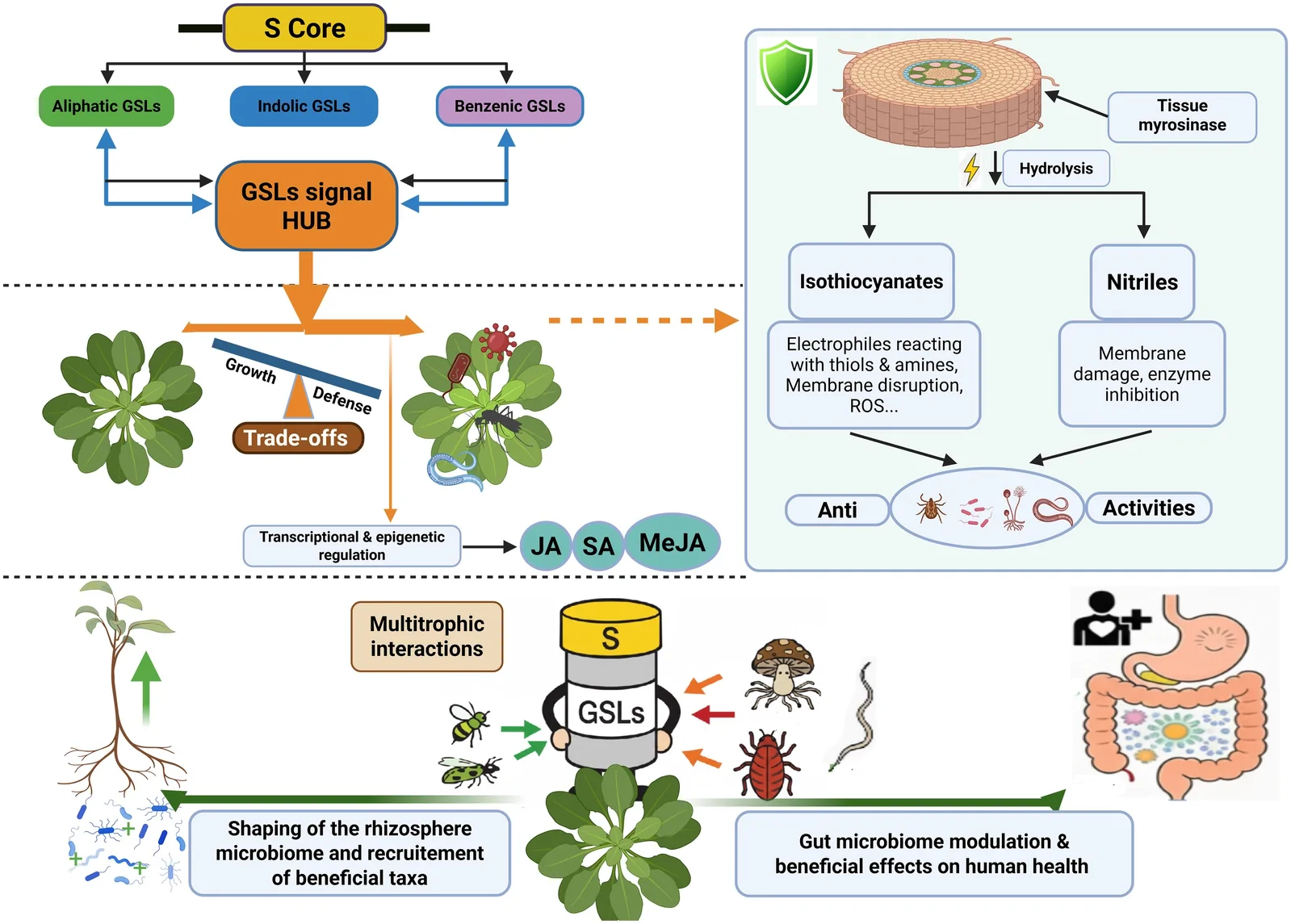
Glucosinolates: a bridge between plant defense against pathogens, soil microbiome modulation, and human health. S-containing glucosinolates (GSLs) play a crucial role in plant defense and the maintenance of plant health. The first panel shows the three main classes of glucosinolates: aliphatic, indolic, and aromatic. The second panel depicts the trade-offs between resources being preferentially allocated to GSLs in defense against pathogens and herbivores and growth under biotic stress. This reallocation is accompanied by an enhanced expression of genes involved in glucosinolate biosynthesis, thereby influencing the transcriptional and epigenetic regulation of defense responses. In the GSL-based defense mechanisms, glucosinolates are first transported to sites of infection or attack and then hydrolyzed by tissue myrosinase upon cell damage (shown in the third panel). This hydrolysis generates biologically active compounds which exert various defensive activities. In addition, the last panel illustrates the key role of GSLs in multitrophic interactions, modulating plant–insect–pathogen relationships, and in structuring of the rhizosphere microbiome by promoting the recruitment of beneficial microorganisms. Finally, the consumption of glucosinolate-rich plants is associated with modulation of the gut microbiome and with beneficial effects on human health. Created in BioRender. Türksoy, G.M. (2026) https://BioRender.com/i4yiva2.

Organosulfur compounds in *Allium* species, such as garlic or onion, act as S-containing phytoanticipins, similar to glucosinolates. These species produce amino acid-based metabolites, such as alliin, isoalliin, and methiin, and store them in the cytosol (Abdalla and Mühling, 2019; Yoshimoto *et al*., 2022). Upon cellular damage, these compounds are metabolized by alliinase, a cysteine sulfoxide lyase normally localized in the vacuole. The main product formed is allicin, derived from alliin, which has direct anti-microbial effects, due to its redox activity and ability to easily penetrate cell membranes. Allicin can also rearrange into various S-VOCs, such as diallyl sulfides, dithiins, and ajoene (Nwachukwu *et al*., 2012). Alliinase is not specific to *Allium* species; it is also found in the medicinal herb *Petiveria alliacea*, where it activates petiveriin into phenylmethanesulfenic acid (PMSA). This compound can be further converted into thiosulfinate petivericin or phenylmethane thial-*S*-oxide (Musah *et al*., 2009). These and other S-containing compounds from *P. alliacea* exhibit biological activity against various pathogenic fungi and bacteria (Kim *et al*., 2006).

S-containing phytoalexins are the next major functional class of defense metabolites. Indolic phytoalexins specific to the Brassicales, such as camalexin, brassinin, and cyclobrassinin, have been well studied and also possess health-promoting activities (Chripkova *et al*., 2014; Abdalla and Mühling, 2019). However, not all S-containing phytoalexins contain the indole group, as seen, for instance, in nasturlexins (Fig. 1) (Pedras and To, 2015). In Arabidopsis and *Camelina sativa*, camalexin (Fig. 1) is synthesized from tryptophan through a series of reactions catalyzed by P450 enzymes (Bednarek, 2012; for details on camalexin function, see below). In other *Brassica* species, brassinin is derived from the degradation product of glucobrassicin, indol-3-ylmethyl-isothiocyanate (ITC) (Klein and Sattely, 2017). Elemental sulfur (S^0^) is also a phytoalexin, produced in economically important plants from various plant families. It is highly toxic to several ascomycetes, effectively controlling major diseases such as powdery mildew (Cooper and Williams, 2004).

The two S-containing amino acids, Cys and Met, are essential for maintaining plant health and activating defense mechanisms (Moormann *et al*., 2022). Cys residues are major targets for oxidative post-translational modifications, such as sulfenylation, *S*-nitrosylation, and disulfide bond formation. Being reversible, they fine-tune the activity of core immune proteins, including NADPH oxidases, cysteine-rich receptors, and kinases involved in pathogen-associated molecular pattern (PAMP) perception, making Cys-centered redox control a core element of plant immunity (Couturier *et al*., 2013). In Arabidopsis, Cys acts as both a metabolic precursor and an independent signaling molecule. Exogenous Cys application induces proteome responses similar to those caused by biotic stress, increases levels of the phytoalexin camalexin, and enhances resistance to *Pseudomonas syringae* pv. tomato DC3000 (Moormann *et al*., 2025). Thus, Cys serves as a precursor for secondary metabolites, but also contributes to a metabolic communication system that integrates danger perception, redox signaling, and defense activation, enhancing plant immunity. Cys and GSH play crucial roles in plant defense against fungal and bacterial pathogens by modulating redox homeostasis. After fungal infections by *Blumeria graminis*, an accumulation of GSH in the apoplast triggers resistance signaling, whereas mitochondrial GSH depletion in Arabidopsis leaves upon *B. cinerea* infection leads to reactive oxygen species (ROS)-mediated cell death that restricts pathogen spread (Simon *et al*., 2013). Additionally, Cys-rich proteins, such as membrane-associated thionins, exhibit strong antifungal activity against *Cladosporium cucumerinum* and *B. cinerea*, and are toxic to lepidopteran larvae such as *Helicoverpa* spp. (Kunstler *et al*., 2020).

Also Met plays a crucial role in coordinating metabolism and immune responses in plants. Its derivative, *S*-adenosylmethionine (SAM), is a key precursor of ethylene and polyamines, two major defense signaling molecules (Sauter *et al*., 2013). The Met cycle helps to balance growth and defense in plants. Marco *et al*. (2014) showed that overexpression of SAM decarboxylase 1 (SAMDC1) in Arabidopsis elevated spermine levels and constitutively up-regulated defense genes and jasmonic acid biosynthesis genes, conferring enhanced resistance to *P. syringae* pv. tomato DC3000 and pv. *maculicola* ES4326, and *Hyaloperonospora arabidopsidis* without growth penalties (Marco *et al*., 2014). Additionally, the methionine cycle is critical for antiviral resistance, and its modulation can increase viral susceptibility (Makinen and De, 2019).

The importance of S metabolism in enhancing immunity has been further demonstrated by *in planta* bacterial transcriptomics. During the early stages of pattern-triggered immunity, virulence genes were induced in *P. syringae* pv. tomato DC3000, but in later stages genes related to S starvation response were activated (Lovelace *et al*., 2018). Moreover, upon pathogen inoculation, plants induced transcription of genes for sulfate transporters, indicating a mechanism whereby plants withdraw sulfate from the apoplast to limit bacterial growth in leaves. In this respect, S resembles iron, which is a common subject of the tug of war between the host and the pathogen (Gerner *et al*., 2020).

Altogether, these mechanisms explain the empirical S-induced resistance in crops (Bloem *et al*., 2015). However, there is a metabolic cost associated with the S-mediated defense, highlighting trade-offs between immunity, growth, and symbiotic interactions. S is preferentially allocated to primary assimilation in the morning and to the glucosinolate pool in the evening (Huseby *et al*., 2013), supporting the idea of growth versus defense trade-off. The metabolic costs can be minimized by the activation of S-rich pathways only as a consequence of biotic stress, such as for the S-containing phytoalexins, or by a regulatory interconnection with primary S metabolism (Malhotra *et al*., 2023). Indeed, glucosinolates serve as a pool of S which can be recycled into primary products during seed germination and seedling establishment under limited S supply (Sugiyama *et al*., 2021). S deficiency induces accumulation of Sulfur Deficiency Induced (SDI1) and SDI2 proteins, which repress the transcription of genes involved in aliphatic glucosinolate synthesis (Aarabi *et al*., 2016). This reallocation of S towards primary metabolism compromises chemical defense but enhances protein synthesis. In line with this, pathogen-challenged S-limited Arabidopsis plants show compromised resistance to *Alternaria brassicicola* (Kruse *et al*., 2007). Collectively, these findings position S metabolism as a central resource allocation hub, mediating strategic trade-offs between growth, immunity, and symbiotic benefits under varying nutrient and biotic conditions.

### Glucosinolates, central sulfur metabolites of the Brassicales, in plant–microbe interactions

Glucosinolate biosynthesis is strongly affected by environmental conditions, allowing plants to adjust defenses against biotic stresses and shape rhizosphere microbiome composition (Fig. 3) (Mitreiter and Gigolashvili, 2021; Kattel and Antonious, 2025). Upon pathogen attack, local biosynthesis of 4-methoxyglucobrassinin (4-methoxyindol-3-ylmethyl glucosinolate) is activated to prioritize defense mechanisms (Bednarek *et al*., 2009). This response is orchestrated by complex transcriptional and epigenetic regulation as well as redistribution through specialized membrane transporters, to optimize concentrations at infection sites (Xu *et al*., 2016). The transcriptional regulation is primarily dependent on two groups of transcription factors, MYB28/29/76 for aliphatic glucosinolates and MYB51/34/122 for indolic glucosinolates, which require interaction with MYC2/3/4, connecting the regulation to jasmonate response (Schweizer *et al*., 2013). Numerous studies emphasize glucosinolate biosynthesis as a key pivot in plant–pathogen co-evolution, tightly linked to S metabolism modulation, hormonal defense regulation (especially jasmonate and salicylate), and integration of biotic signals (Fig. 3) (Cacho *et al*., 2015; Kopriva and Gigolashvili, 2016; Liu *et al*., 2016; Sugiyama *et al*., 2021). Natural genetic variation in glucosinolate production under biotic stress is well documented in *A. thaliana*, where polymorphisms in genes such as *AOP2*, *MYB28*, and *MAM3* determine aliphatic glucosinolate profiles, influencing pathogen and herbivore resistance through genotype–environment interactions (Chan *et al*., 2011; Kerwin *et al*., 2015). Transcriptomic studies revealed significant enrichment of transcription of glucosinolate biosynthesis genes and insect response pathways during infection, and highlighted regulation by biotic stress-related phytohormones (Liu *et al*., 2024). Accordingly, proteomic analysis revealed that in Arabidopsis leaves, methyl jasmonate (MeJA) up-regulates oxidative stress tolerance, glucosinolate metabolism, S assimilation, and defense pathways, while it represses photosynthesis and carbohydrate anabolism, thereby redirecting resources from growth to defense (Chen *et al*., 2011). These changes, coupled with root defense priming, maintain glucosinolate profile variation during attacks.

Glucosinolates themselves do not have direct antimicrobial activity; the antifungal, antibacterial, and anti-oomycete effects are attributed to their breakdown products, particularly ITCs (Bednarek *et al*., 2009; Clay *et al*., 2009; Fan *et al*., 2011). The details on the multifaceted function of glucosinolates in pathogen defense, their target organisms, mechanisms, and ecological significance can be found in multiple reviews and the references therein (Kunstler *et al*., 2020; Plaszko *et al*., 2021; Z. Liu *et al*., 2021; Eugui *et al*., 2022; W. Yang *et al*., 2024; Somssich *et al*., 2025). In short, the various glucosinolate degradation products act through multiple mechanisms, including disrupting cell membranes, triggering ROS accumulation, inhibiting enzymes, altering surface hydrophobicity, inhibiting motility, and repelling pathogens.

Besides their role in defense, glucosinolates and their breakdown products belong to an increasing number of plant secondary metabolites that are associated with shifts in the composition of plant-associated microbiota (Jacoby *et al*., 2021). For example, community structures in the rhizosphere changed when exposed to the degradation products of an additionally produced glucosinolate sinalbin (*p*-hydroxybenzyl glucosinolate) in a transgenic Arabidopsis overexpressing CYP79A1 (Bressan *et al*., 2009). Similarly, changes in rhizosphere bacterial community composition of *Brassica napus* correlated with the soil concentration of 2-phenylethyl-ITC, which is derived from gluconasturtiin (phenethyl glucosinolate; Rumberger and Marschner, 2004). Atypical myrosinases such as the PYK10 (BGLU23) and BGLU21 localized in endoplasmic reticulum (ER) bodies contribute to the production of bioactive metabolites from glucosinolates as evidenced from experiments with the Arabidopsis double mutant *pyk10 bglu21* that showed altered bacterial and fungal root-associated communities compared with the wild type (Basak *et al*., 2024). Similar alterations were observed in the *nai1* mutant, which lacks functional ER bodies, confirming the importance of these structures for Arabidopsis interaction with microbiota and suggesting that glucosinolate degradation occurs before root exudation (Basak *et al*., 2024). The myrosinase reactions can have diverse outcomes, depending on the presence of specifier proteins directing the reaction toward nitriles, epithionitriles, and thiocyanates (Kissen and Bones, 2009). Manipulation of the degradation pathway in the Arabidopsis triple *nsp* mutant, which does not produce nitriles, also caused distinct shifts in rhizosphere β-diversity, which was distinct from mutants impaired in glucosinolate biosynthesis (Chroston *et al*., 2024). These findings highlight that not only glucosinolates *per se*, but also the specific myrosinases and the type of glucosinolate hydrolysis products contribute to the assembly of root-associated microbial communities. Additionally, specific glucosinolates can enrich certain microbial taxa in leaf communities (Unger *et al*., 2024). Analyses of wild Arabidopsis populations revealed that sinigrin (allyl-glucosinolate) enriches specific taxa because they can use it as a carbon source, in contrast to glucoraphanin (4-methylsulfinylbutyl glucosinolate). Degrading sinigrin, certain strains of the *Comamonadaceae* and *Oxalobacteraceae* strains cross-feed other taxa, enabling formation of complex communities (Unger *et al*., 2024). While the role of glucosinolates in shaping plant-associated microbiota is increasingly recognized, similar principles appear to operate in animal systems.

Plant-derived glucosinolates and ITCs also impact the composition and function of the animal gut microbiota, as the animals feed on glucosinolate-producing plants. They enhance bacterial diversity, stimulate production of beneficial microbial metabolites, and modulate host immune responses, leading to anti-inflammatory and anticarcinogenic effects (Sikorska-Zimny and Beneduce, 2021). The most frequently studied dietary glucosinolates involved in these modulations are glucoraphanin and glucobrassicin (indol-3-ylmethyl glucosinolate). When plant myrosinase is inactivated through cooking, the gut microbiota can partially compensate by converting glucosinolates into bioactive ITCs, such as sulforaphane [4-(methylsulfinyl)butyl-ITC], through myrosinase-like activity (Dmytriv *et al*., 2025). Dietary supplementation with glucoraphanin in obese C57BL/6J mice fed a high-fat diet reduced body weight gain and improved glucose tolerance, compared with control mice (Bankole *et al*., 2024). This was accompanied by a restoration of gut eubiosis, a significant decrease in the Firmicutes/Bacteroidetes ratio, a hallmark of obesogenic dysbiosis, and broad remodeling of microbial diversity, community composition, and the plasma metabolome (Bankole *et al*., 2024). Controlled human intervention studies have shown that broccoli consumption alters gut microbiota β-diversity, reduces accumulation of Firmicutes, and increases Bacteroidetes, resulting in a lower Firmicutes/Bacteroidetes ratio, similar to glucoraphanin supplementation (Kaczmarek *et al*., 2019). Indeed, these shifts correlated with accumulation of glucosinolate-derived metabolites in plasma and urine. Consistently, *in vitro* experiments with human fecal microbiota fed broccoli extracts confirmed that the microbiota broke down glucosinolates, and corresponding ITCs accumulated in the extract (Zhang *et al*., 2022). Moreover, feeding the extract increased microbial diversity and caused marked restructuring of microbial communities (Zhang *et al*., 2022). While dietary glucosinolates are clearly implicated in shaping gut microbiota composition and function, most evidence derives from whole-food extracts or *in vitro* models. More research is needed to understand the specific effects of individual glucosinolates on the gut microbiota (Sikorska-Zimny and Beneduce, 2021). Additionally, controlled animal studies are necessary to disentangle the variability driven by microbiota and to elucidate the mechanisms underlying glucosinolate–microbiome–health interactions.

Further investigations are needed to assess the mechanisms by which glucosinolates and their degradation products shape microbiomes in the plant and animal kingdoms. Comparable effects of glucosinolates and derived products have been observed in the microbiota of plant rhizospheres and animal guts, suggesting that myrosinase-mediated degradation plays a key role in the interaction of glucosinolate-producing plants with their associated microbes as well as in effects on intestinal microbiota upon glucosinolate consumption by animals. Glucosinolate production and degradation have been linked to a decreased abundance of opportunistic taxa, such as *Pedobacter*, *Caulobacter*, *Hirschia*, and *Ferruginibacter*, and an enrichment of beneficial taxa including *Pseudoxanthomonas* and *Brevundimonas* (Chroston *et al*., 2024), similar to improvement of the Firmicutes/Bacteroidetes ratio in the gut microbiome after consuming broccoli extract (Zhang *et al*., 2022; Bankole *et al*., 2024). Together, these findings suggest that glucosinolates help to shift structurally and functionally dysbiotic communities towards more balanced microbial assemblages that benefit host health and ecosystem functioning. Although the underlying molecular mechanisms are not fully understood, these convergent outcomes highlight glucosinolates as promising cross-kingdom modulators of microbial community structure, stability, and function. Further research integrating isotope labeling, gnotobiotic systems, and genetic tools is needed to establish causality in these interactions, beyond correlative association.

### Camalexin, a model S-containing phytoalexin

Camalexin was first isolated in *Camelina sativa*, and has since been widely studied for its antimicrobial effect against pathogens such as *B. cinerea*, *P. syringae*, *Verticillium dahliae*, *Plasmodiophora brassicae*, *A. brassicicola*, or *Burkholderia glumae* (Rogers *et al*., 1996; Nguyen *et al*., 2022). Loss of camalexin biosynthesis compromises resistance and, notably, can convert otherwise beneficial interactions into dysbiotic outcomes, as demonstrated for the endophytic fungus *Piriformospora indica*, and oomycetes in multi-kingdom SynComs (Nongbri *et al*., 2012; Wolinska *et al*., 2021). Camalexin acts as a phytoalexin, so that its biosynthetic pathway is activated upon recognition of microbe-associated molecular patterns (MAMPs) (Fig. 4) (Finnegan *et al*., 2016). After recognition of pathogens, calcium bursts activate Ca^2+^-dependent protein kinases (CPKs), CPK5 and CPK6, while mitogen-activated protein (MAP) kinase cascades activate MPK3 and MPK6. These kinases converge on the transcription factor WRKY33, the master regulator of camalexin synthesis genes (Zhou *et al*., 2020). Hormonal signals, particularly jasmonate and ethylene, further reinforce this transcriptional activation through interaction with ERF and MYB transcription factors, such as ERF1, ERF72, MYB51, and MYB122 (Frerigmann *et al*., 2015; Zhou *et al*., 2020, 2022; D. Wang *et al*., 2020; Li *et al*., 2022).

**Fig. 4.**
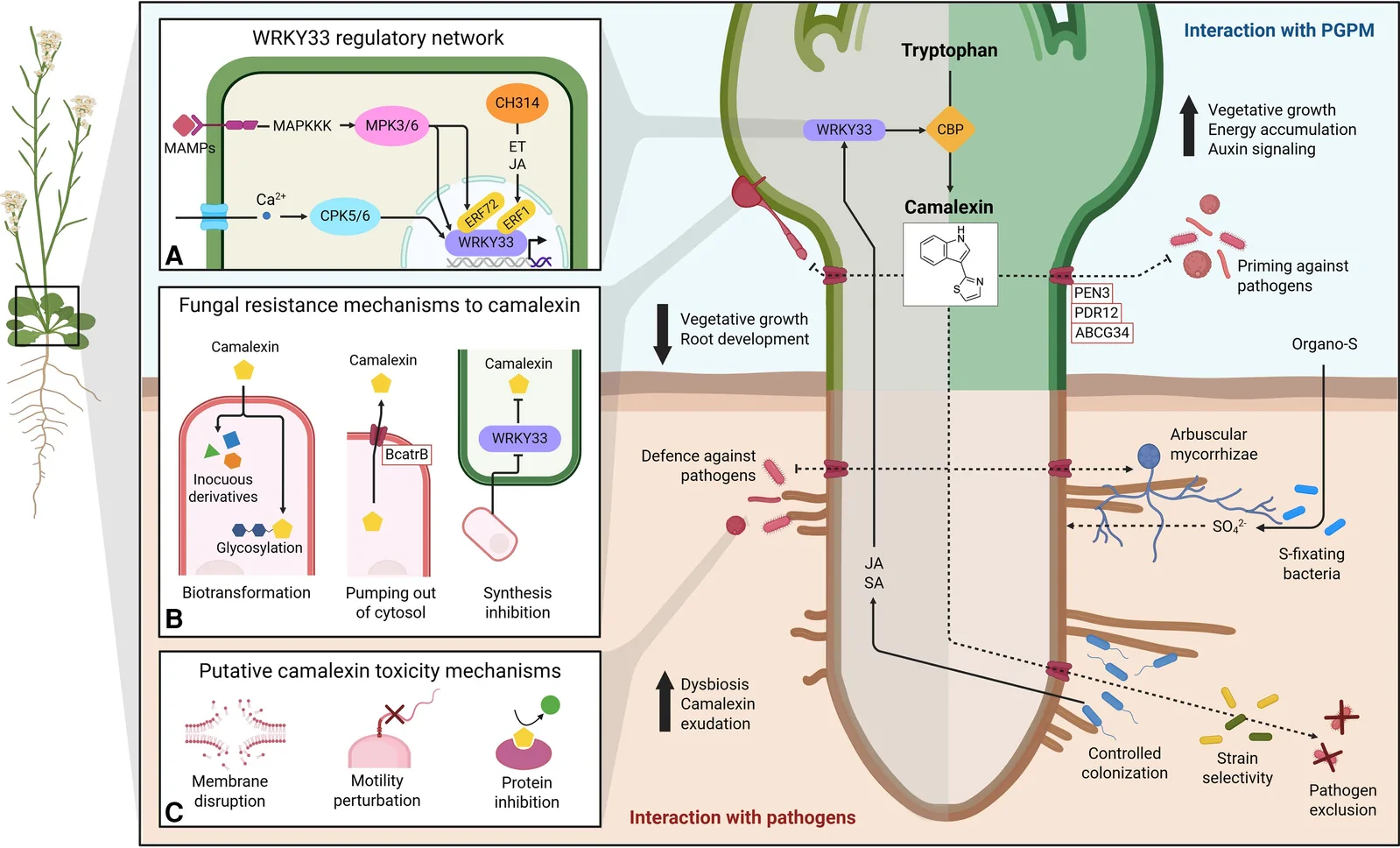
Roles and mechanisms of action of camalexin in the interaction of *A. thaliana* with microbes. Beneficial microbes are drawn in blue and pathogens are drawn in red. Solid arrows represent known pathways, and dashed arrows represent proposed or not fully defined pathways. Interactions with pathogens are shown at the left side of the main panel, and interactions with beneficial microbes are shown at the right side. Internal panels show (A) the regulatory network of the master phytoalexin transcription factor WRKY33 upon sensing of microbe-associated molecular patterns (MAMPs), (B) the known camalexin resistance and detoxification mechanisms of select fungal pathogens, and (C) the hypothesized toxicity mechanisms of camalexin against bacterial and fungal pathogens. Abbreviations: PGPM, plant growth-promoting microbes; CBP, camalexin biosynthetic pathway. Created in BioRender. Elkatmis, B. (2026) https://BioRender.com/j2ubbp3.

Beyond transcriptional regulation, recent evidence demonstrates epigenetic fine-tuning of camalexin biosynthetic genes. A bivalent chromatin state marked by the repression mark H3K27me3 and the activation mark H3K18ac modulates the timing and amplitude of the camalexin biosynthetic gene activation following elicitation (Zhao *et al*., 2021). In addition, core biosynthetic enzymes CYP79B2, CYP71A12, CYP71A13, GSTU4, and CYP71B15 assemble into a metabolic complex at the ER surface that allows channeling of tryptophan and its byproducts without the accumulation of intermediates (Mucha *et al*., 2019). While the precise mechanisms are still being studied, these discoveries pinpoint the complex multilevel regulatory mechanisms and the importance of induction–repression balance for the observed time-specific camalexin response against distinct pathogens. Due to its early discovery and prominent presence in the model species Arabidopsis, the camalexin-mediated immune response to pathogens is well understood and amply evidenced. Most new studies focus not on its antimicrobial function, but rather on the regulatory landscapes underlying the biosynthetic route activation, metabolite mobilization, and its relationship with the nutritional status of the plant.

Although the protective function of camalexin is well established, its precise modes of toxicity vary among target organisms and are not yet comprehensively understood. Proposed mechanisms include: (i) disruption of membrane integrity via binding and destabilization of the phospholipid bilayer (Fig. 4) observed in *P. syringae* and *P. simiae* (Rogers *et al*., 1996); (ii) inhibition of bacterial motility and chemotaxis implied by transcriptomics (J. Zhou *et al*., 2025); (iii) interference with protein function, such as binding to and inhibiting the AcrB efflux pump in *Escherichia coli* (Irianti *et al*., 2025); and (iv) inhibition of viral particle assembly (Liao *et al*., 2022). In response, distinct fungal pathogens have evolved various strategies to handle camalexin toxicity. Several species, including *Rhizoctonia solani*, *Sclerotinia sclerotiorum*, *Leptosphera maculans*, *A. brassicicola*, and *B. cinerea*, perform biotransformations, during which they metabolize camalexin into innocuous or less toxic derivatives by oxidation, hydrolysis, or glycosylation of the original compound (Fig. 4) (Pedras and Abdoli, 2013). Other strategies, found in *B. cinerea*, include the reduction of camalexin synthesis in the host by dampening the expression of the master regulator WRKY33 (Liu *et al*., 2017), as well as the expression of the ABC transporter BcatrB, which allows the active pumping of camalexin out of the fungal cytosol (Stefanato *et al*., 2009), effectively reducing its cytotoxic effect. Overall, while these reciprocal adaptations highlight camalexin as a focal point of plant–pathogen co-evolution, the specific mechanistics behind these effects remain understudied. Defining the particular response mechanisms in bacteria and fungi is essential for a full understanding of the plant–pathogen communication system.

Beyond its antimicrobial function, camalexin has a dual role in shaping plant microbiome composition and function (Koprivova *et al*., 2019). A genome-wide association study identified a tandem of two Arabidopsis P-450 genes, *CYP71A27* and *CYP71A28*, that contribute to control of variation in sulfatase activity as a proxy for microbiome function (Koprivova *et al*., 2019). Mutants in core camalexin synthesis genes phenocopied the *cyp71A27* mutant and camalexin complemented the reduction in soil sulfatase activity. Interestingly, camalexin was also found to be necessary for the PGP effect of beneficial strains, such as *Paraburkholderia phytofirmans* PsJN, *Pseudomonas* sp. CH267, and *Pseudomonas simiae* WCS417r (Koprivova *et al*., 2019). This is consistent with camalexin exudation from roots being triggered by elicitors (Millet *et al*., 2010). Interestingly, camalexin exuded from roots is synthesized in the leaves, implying systemic coordination between above-ground defense perception and below-ground microbiome regulation (Koprivova *et al*., 2023). This challenges the traditional view of phytoalexins as strictly local defense compounds and positions camalexin as a systemic mediator of microbial balance. Taken together, this evidence suggests that rather than acting solely as a toxin, camalexin appears to prevent microbial overproliferation and dysbiosis, thereby stabilizing beneficial associations.

Additionally, camalexin is part of the priming mechanism for protection against pathogen infection, known as induced systemic resistance (ISR) (Bakker *et al*., 2018). For instance, root colonization by *Trichoderma virens* and *T. atroviride* enhanced growth and induced the expression of defense pathways and camalexin accumulation, which reduced the severity of infection by *B. cinerea* (Contreras-Cornejo *et al*., 2011). Similarly, root colonization by the rhizobacterium *Bacillus cereus* AR156 activated genes for camalexin synthesis, effectively accumulating camalexin, which provided Arabidopsis with a wide-spectrum resistance against leaf infection by different pathogens (Li *et al*., 2024). Camalexin-mediated ISR against *B. cinerea* was also primed by the phyllospheric yeast *Protomyces arabidopsidicola* strain C29 (Wang *et al*., 2022). Camalexin and other tryptophan-derived compounds are also important for the ability of commensal bacteria to control fungal load in the rhizosphere, thereby promoting Arabidopsis growth and survival (Wolinska *et al*., 2021). This evidence shows that PGP colonization further benefits Arabidopsis by allowing it to turn post-infection camalexin synthesis into a constitutive resistance factor that represents an ample ecological advantage.

Taken together, camalexin exemplifies the multifunctionality of S-containing defense metabolites. It operates at the intersection of pathogen restriction, microbiome assembly, systemic signaling, and growth–defense balance. Rather than acting solely as a cytotoxic endpoint of immunity, camalexin appears to modulate microbial community dynamics in a manner that prevents dysbiosis while preserving beneficial interactions. Understanding how camalexin concentration, spatial distribution, and metabolic context shape these outcomes remains an important challenge.

## The role of plant sulfur metabolism in interactions with beneficial microbes under abiotic stress

Many PGP bacteria enhance plant performance specifically under abiotic stress conditions (Muhammad *et al*., 2024). They employ various mechanisms to support plants under unfavorable environments, such as modulating phytohormones, enhancing nutrient uptake, activating systemic resistance against pathogen infection, and boosting antioxidant mechanisms in plants (Backer *et al*., 2018; Muhammad *et al*., 2024). For example, many PGP strains produce 1-aminocyclopropane-1-carboxylate (ACC) deaminase, an enzyme that breaks down ACC to reduce stress-induced ethylene production in plants, thereby alleviating stress symptoms (C.-H. Liu *et al*., 2021; Shekhawat *et al*., 2023). Additionally, certain PGP bacteria modulate enzymatic antioxidants such as superoxide dismutase, catalase, and ascorbate peroxidase, as well as non-enzymatic antioxidants, such as flavonoids, carotenoids, and GSH, to strengthen the defense of the plant under abiotic stress conditions (Q. Wang *et al*., 2020; Tian *et al*., 2025). Thus, beneficial microbes play a critical role in mitigating abiotic stress, and S-containing compounds, such as GSH, are often part of these mechanisms (Fig. 5) (Noctor and Foyer, 1998).

**Fig. 5.**
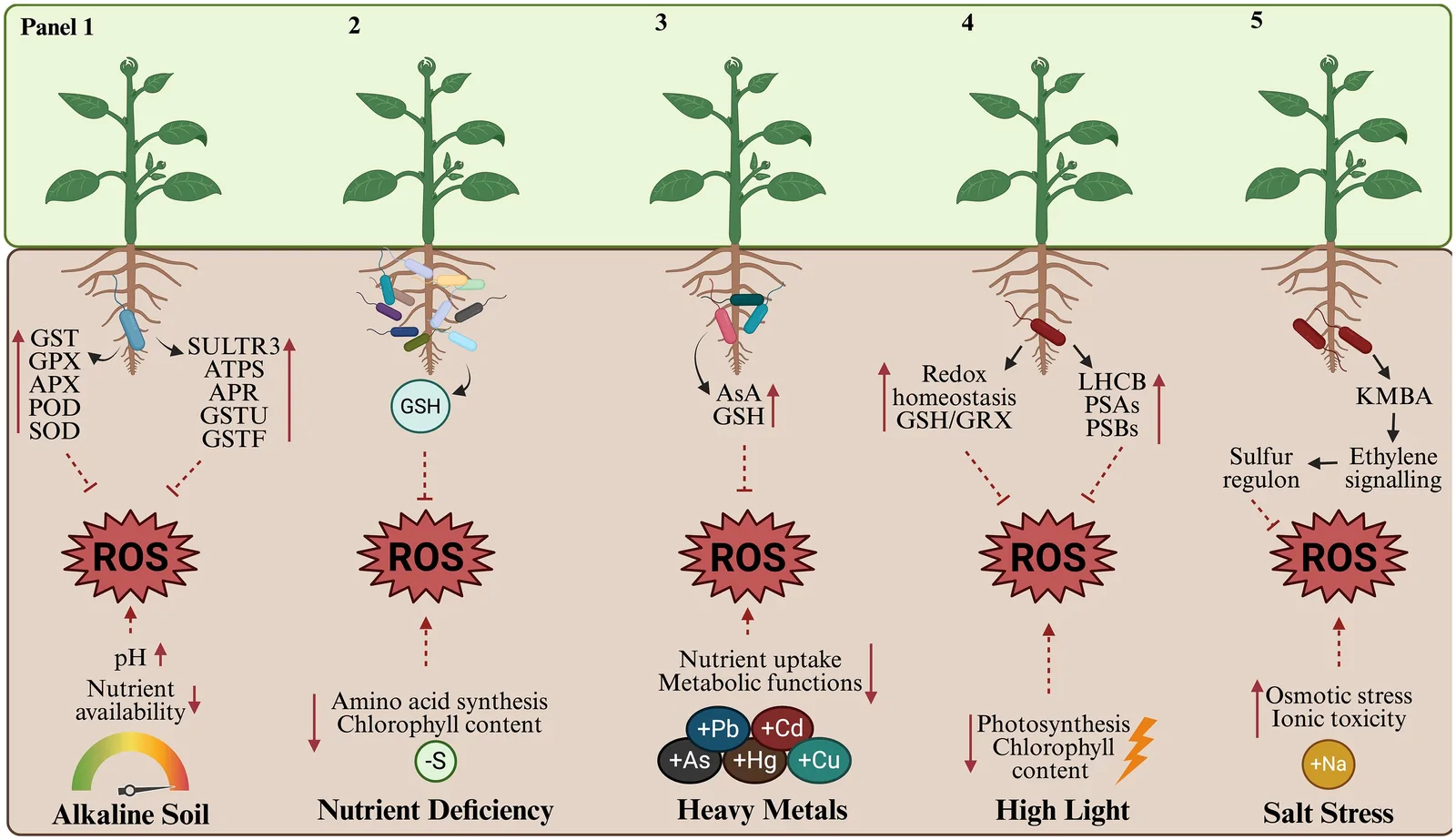
Mechanisms employed by beneficial microbes to modulate S metabolism under abiotic stress. This figure summarizes some of the S-related mechanisms employed by beneficial microbes to mitigate abiotic stress-induced oxidative damage in plants. Panel 1. Alkaline stress increases soil pH, reduces nutrient availability, and enhances ROS accumulation. *Bacillus* sp. Jrh14-10 enhances the expression of S assimilation-related genes and antioxidant enzymes. Panel 2. Sulfur deficiency. Low S availability reduces amino acid biosynthesis and chlorophyll content. An 18-member SynCom secretes extracellular GSH, thereby alleviating the effects of S deficiency. Panel 3. Heavy metal stress impairs nutrient uptake and metabolic functions. Inoculation with three metal-resistant bacterial strains increases ascorbic acid (AsA) and GSH levels in *Brassica napus* grown in copper-contaminated soil, thereby reducing ROS-induced damage. Panel 4. Light stress reduces photosynthetic efficiency and chlorophyll content. *Enterobacter* sp. SA187 enhances redox homeostasis by modulating the GSH/glutaredoxin (GRX) balance and regulating the expression of photosynthesis-related genes, including LHCB (light-harvesting chlorophyll *a*/*b*-binding protein), PSAs (PSI subunits), and PSBs (PSII subunits). Panel 5. Salt stress induces ionic toxicity, leading to elevated ROS levels in plants. SA187 regulates ethylene signaling via 2-keto-4-methylthiobutyric acid (KMBA), activating the sulfur regulon and protecting plants from ROS-associated damage. Created in BioRender. Elkatmis, B. (2026) https://BioRender.com/s86ph1e.

Indeed, S has irreplaceable functions in the plant antioxidant system, being essential for the synthesis of key molecules such as GSH, Cys, Met, and others that help to maintain redox balance and protect against abiotic stress (Kopriva *et al*., 2019). GSH is synthesized through a two-step process catalyzed by γ-glutamylcysteine synthetase and glutathione synthetase (Noctor *et al*., 2012). GSH is easily oxidized by, for example, ROS to glutathione disulfide (GSSG) and is thus essential for cellular redox homeostasis (Noctor and Foyer, 1998; Noctor *et al*., 2012). Various GSH-dependent proteins and enzymes, including glutathione peroxidases, glutaredoxins, and glutathione *S*-transferases (GSTs) further contribute to antioxidant defense in plants (Dixon *et al*., 2009; Cao *et al*., 2020; Vogelsang and Dietz, 2022). Recent studies revealed that beneficial microbes not only influence plant antioxidant systems but also impact redox homeostasis through S metabolism and GSH-dependent pathways, aiding plants in coping with abiotic stress (Guo *et al*., 2023; Shekhawat *et al*., 2024; Bisht *et al*., 2025).

In a typical example, a root endophytic bacterium *Enterobacter* sp. SA187 enhances plant growth under various abiotic stress conditions through multiple mechanisms. It modulates ethylene signaling by producing 2-keto-4-methylthiobutyric acid that is converted to ethylene (de Zelicourt *et al*., 2018). Additionally, after colonizing plants, SA187 strongly up-regulates genes related to S metabolism (Andres-Barrao *et al*., 2021). This helps to rescue salt hypersensitivity of Arabidopsis mutants in S metabolism, such as *sultr1;2*, *apr2*, or *apk1 apk2*, and indicates that coordinated regulation of S metabolism between plants and PGP microbes enhances plant salt stress tolerance (Andres-Barrao *et al*., 2021). SA187 also improves plant tolerance to high light stress by modulating iron and S acquisition (Shekhawat *et al*., 2024), leading to increased expression of genes related to photosynthesis and oxidative stress responses. Using a GRX1/ro-GFP2 reporter line, Shekhawat *et al*. (2024) demonstrated that SA187 strengthened redox homeostasis under high-light conditions through regulating iron homeostasis and redox balance.

Another common stress is high alkalinity, which alters water absorption, nutrient availability, and soil pH, impairing plant growth and development (S. Yang *et al*., 2024). Inoculation with *Bacillus* sp. Jrh14-10 enhanced Arabidopsis growth under high alkalinity stress by increasing total fresh weight, dry weight, primary root length, and lateral root number (Guo *et al*., 2023). Interestingly, similar positive effects were observed after exogenous addition of MgSO_4_, Cys, and GSH. Indeed, transcriptome analysis revealed that Jrh14-10 treatment up-regulated S metabolism genes, including *APS3*, *APR1*, *APR2*, *APR3*, *SERAT3;2*, and *OASC*, leading to increased production of Cy and other S-containing metabolites (Guo *et al*., 2023). Moreover, Jrh14-10 treatment reduced ROS levels under high alkalinity by enhancing activities of antioxidant enzymes (Guo *et al*., 2023).

Furthermore, PGP bacteria can improve plant performance under heavy metal stress (Ren *et al*., 2019). Inoculation with metal-resistant strains *Microbacterium oxydans* JYC17, *Pseudomonas thivervalensis* Y1-3-9, and *Burkholderia cepacia* J62 increased the biomass of *B. napus* L. grown in copper-contaminated soil and enhanced the levels of antioxidants ascorbic acid (AsA) and GSH in plant leaves. These two antioxidants are key components of the AsA–GSH cycle, which detoxifies hydrogen peroxide (Noctor and Foyer, 1998). The increased AsA and GSH levels following bacterial inoculation suggest that beneficial microbes may enhance antioxidant capacity to mitigate oxidative damage caused by heavy metal toxicity (Ren *et al*., 2019). In nature, however, plants are often exposed to multiple abiotic stresses, which intensifies their impact on growth. In such conditions, PGP bacteria can provide the greatest benefit. For instance, treatment with *Bacillus amyloliquefaciens* SN13 increased the growth of rice under drought, salt stress, and nutrient deficiency, by modulating antioxidant enzyme activities and reducing oxidative stress in plants (Bisht *et al*., 2025). While these studies highlight the importance of S metabolism in plant–microbe interactions under abiotic stress, further reserach is needed to directly demonstrate the contribution of beneficial bacteria to plant S uptake, transport, and metabolic integration.

## Conclusions and future perspectives

Across nutritional, defensive, and ecological contexts, S emerges as a central integrator of plant–microbe interactions. S-containing compounds function as nutrients, redox regulators, antimicrobial agents, long-distance signals, and microbiome-shaping metabolites. This multifunctionality enables S metabolism to coordinate growth, immunity, and ecological balance under fluctuating environmental conditions. Despite substantial progress, several key gaps remain. First, the quantitative contribution of microbiota to plant S nutrition is largely inferred from transcriptional and metabolomic data rather than directly measured. Stable isotope-based S flux analyses are urgently needed to resolve microbial-to-plant S transfer under ecologically relevant conditions. Second, while the biosynthesis of S-VOCs is increasingly understood, their perception mechanisms in plants remain largely unknown. Identification of receptors and downstream signaling components represents a major frontier. A broader conceptual challenge lies in integrating S metabolism into systems-level models of plant immunity and microbiome assembly. S allocation is tightly balanced between primary metabolism and defense, reflecting trade-offs between growth and protection. Understanding how plants dynamically regulate this allocation in response to microbial signals and environmental stress will be critical for predictive modeling and crop improvement. Future research combining isotope tracing, synthetic microbial communities, genome editing, and ecological field studies will be instrumental in transforming our current descriptive understanding into a mechanistic framework. Harnessing S metabolism as a target for microbiome engineering and sustainable agriculture represents a promising avenue for improving plant resilience under both biotic and abiotic stress.
